# Hardware Error Correction for MZI-Based Matrix Computation

**DOI:** 10.3390/mi14050955

**Published:** 2023-04-27

**Authors:** Huihuang Hou, Pengfei Xu, Zhiping Zhou, Hui Su

**Affiliations:** 1 Key Laboratory of Optoelectronic Materials Chemistry and Physics, Fujian Institute of Research on the Structure of Matter, Chinese Academy of Sciences, Fuzhou 350002, China; houhuihuang@fjirsm.ac.cn; 2 University of Chinese Academy of Sciences, Beijing 100049, China; 3 State Key Laboratory of Advanced Optical Communication Systems and Networks, School of Electronics, Peking University, Beijing 100871, China; xupengf@pku.edu.cn; 4 Beijing Aijie Optoelectronic Technology Co., Ltd., Beijing 100190, China; 5 Fujian Science & Technology Innovation Laboratory for Optoelectronic Information of China, Fuzhou 350108, China

**Keywords:** hardware error correction, matrix computation, Mach–Zehnder interferometer

## Abstract

With the rapid development of artificial intelligence, the electronic system has fallen short of providing the needed computation speed. It is believed that silicon-based optoelectronic computation may be a solution, where Mach–Zehnder interferometer (MZI)-based matrix computation is the key due to its advantages of simple implementation and easy integration on a silicon wafer, but one of the concerns is the precision of the MZI method in the actual computation. This paper will identify the main hardware error sources of MZI-based matrix computation, summarize the available hardware error correction methods from the perspective of the entire MZI meshes and a single MZI device, and propose a new architecture that will largely improve the precision of MZI-based matrix computation without increasing the size of the MZI’s mesh, which may lead to a fast and accurate optoelectronic computing system.

## 1. Introduction

Benefiting from the era of big data, the rapid growth of the Internet provides sufficient training datasets for functioning artificial intelligence (AI), which has also become a hot spot in the current technological revolution. However, artificial intelligence has conventionally relied on electronic processors, with its computing power being greatly determined by transistor numbers and capabilities; managing the massive amount of data necessitates additional computing resources. In the post-Moore era, the increasing transistor numbers can no longer keep up with the computing power demand of artificial intelligence, and electronic computation is therefore stuck in a bottleneck [1,2,3]. On the other hand, photons are bosons, which have the advantages of a higher transmission speed and can work well with electrons through the Einstein coefficients, which is conducive to the realization of ultra-high-speed optoelectronic computing. Consequently, more and more researchers are beginning to focus on optoelectronic computing [4,5,6,7,8,9].

The fabrication techniques for silicon photonics are advancing rapidly, enabling the creation of large-scale and complicated circuits and paving the way for low-loss and low-cost optoelectronic devices [10]. These devices can be produced using complementary metal–oxide–semiconductor (CMOS) fabs [11]. As a result, silicon-based optoelectronic computation has emerged as a prominent area of research [3]. At present, optoelectronic matrix computation mainly includes three implementation methods [12]: multi-plane light conversion (MPLC), the Mach–Zehnder interferometer method (MZI), and wavelength division multiplexing (WDM). The Mach–Zehnder interferometer method is simple and easy to integrate. It is one of the best choices to implement optoelectronic matrix computation [13].

The optoelectronic matrix computation implemented by the Mach–Zehnder interferometer is a kind of analog computation, in which computational precision is the most important and should be treated with special attention. However, the performance of silicon photonic devices is sensitive to the influence of environmental disturbances [14,15,16,17], fabrication [15,18], and device aging [15,19], including MZI devices. Particularly, the hardware errors generated by fabrication are the focus of research [20,21], which result in deviations between the matrix implemented by MZI and the actual needed matrix. The hardware errors involve beam splitter errors [20], phase errors [22], and errors caused by optical loss differences [23]. The hardware errors will accumulate with the increase of the scale of the MZI meshes, which limits the implementation of large-scale matrix computation based on MZI meshes. In order to overcome this limitation of hardware error, especially beam splitter error, researchers have proposed different MZI meshes or different MZI devices with higher precision.

In this paper, the Mach–Zehnder interferometer method of achieving optoelectronic matrix computation is introduced in Section 2. The origins of the hardware error of the Mach–Zehnder interferometer are analyzed in Section 3, followed by a synthesis of the studies of MZI hardware error correction in Section 4. Finally, a feasible approach to mitigate the MZI hardware error is proposed in Section 5.

## 2. Methods

**Figure 1:**
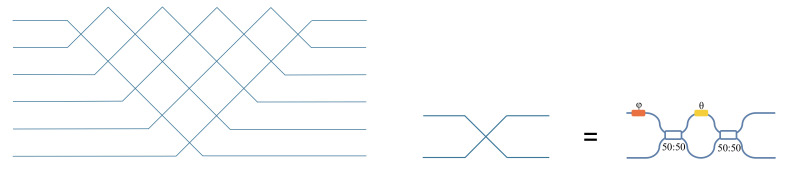
Triangular mesh.

In 1994, Reck et al. [24] proposed one type of triangular mesh based on the Mach–Zehnder interferometer and demonstrated that the unitary matrix transformation of any finite dimension could be implemented by MZI devices. They decomposed the N-dimensional unitary matrix into a series of two-dimensional unitary matrices, which were presented as a triangular mesh formed by the arrangement of MZIs, as shown in Figure 1.

**Figure 2:**
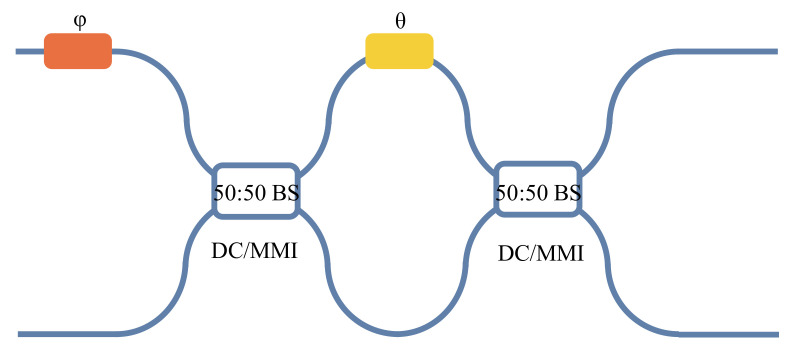
The common MZI.

A common Mach–Zehnder interferometer consists of an external phase shifter (φ), an internal phase shifter (θ), and two 50:50 beam splitters (directional couplers (DC)) or multimode interference couplers (MMI)), as shown in Figure 2.

For an MZI device, transmission matrix *S* is

(1) $$S=C_{4}P_{3}C_{2}P_{1}$$

where *P*_1_ and *P*_3_ are the transmission matrices of the external phase shifter and the internal phase shifter, respectively; *C*_2_ and *C*_4_ are the transmission matrices of the beam splitter on the left side of the MZI and the beam splitter on the right side of the MZI, respectively. Among them, the left and right beam splitters are considered as ideal 50:50 beam splitters. Hence, MZI transmission matrix *S* can be written as [25]

(2) $$S=\left[\begin{array}{cc} 1/\sqrt{2} & i/\sqrt{2}\\ i/\sqrt{2} & 1/\sqrt{2} \end{array}\right]\left[\begin{array}{cc} e^{i\theta } & 0\\ 0 & 1 \end{array}\right]\left[\begin{array}{cc} 1/\sqrt{2} & i/\sqrt{2}\\ i/\sqrt{2} & 1/\sqrt{2} \end{array}\right]\left[\begin{array}{cc} e^{i\varphi } & 0\\ 0 & 1 \end{array}\right]$$

(3) $$S=ie^{i\theta /2}\left[\begin{array}{cc} e^{i\varphi }sin\theta /2 & cos\theta /2\\ e^{i\varphi }cos\theta /2 & -sin\theta /2 \end{array}\right]$$

where *φ* and *θ* are phases produced by the external phase shifter and the internal phase shifter, respectively. Obviously, we can produce different phases through these two phase shifters to implement two-dimensional unitary matrices with different elements. For an N-dimensional unitary matrix, the MZI’s transmission matrix can be generalized as *T*_n,m_ (θ, φ):

(4) $$T_{n,m}\left(\theta ,\varphi \right)=\left[\begin{array}{cccccccc} 1 & 0 & & \cdots & \cdots & & 0 & 0\\ 0 & 1 & & & & & 0 & 0\\ & & \ddots & & & & & \\ \vdots & & & e^{i\varphi }sin\theta /2 & cos\theta /2 & & & \vdots \\ \vdots & & & e^{i\varphi }cos\theta /2 & -sin\theta /2 & & & \vdots \\ & & & & & \ddots & & \\ 0 & 0 & & & & & 1 & 0\\ 0 & 0 & & \cdots & \cdots & & 0 & 1 \end{array}\right]$$

where n and m represent the transmission matrix of the MZI between the nth and mth input ports of the signal entering the mesh. The dimension of an N-dimensional unitary matrix, *U*(*N*), can be reduced by right multiplying *T*_n,m_ (θ, φ), namely,

(5) $$U\left(N\right)T_{N,N-1}T_{N,N-2}\cdots T_{N,1}=\left[\begin{array}{cc} U\left(N-1\right) & 0\\ 0 & 1 \end{array}\right]$$

The N-dimensional unitary matrix’s dimension can be continued to be reduced according to the above dimensionality reduction method, and finally, a diagonal matrix, *D*, with elements of modulo 1 is obtained.

Let

(6) $$R\left(N\right)=T_{N,N-1}T_{N,N-2}\cdots T_{N,1}$$

(7) $$U\left(N\right)R\left(N\right)R\left(N-1\right)R\left(2\right)=\left[\begin{array}{ccccc} 1 & & \cdots & & 0\\ & \ddots & & & \\ \vdots & & 1 & & \vdots \\ & & & \ddots & \\ 0 & & \cdots & & 1 \end{array}\right]=D$$

It can be seen from the previous theoretical derivation that the final implementation of the N-dimensional unitary matrix will be a triangular mesh with N-1 MZIs in the N row and N-2 MZIs in the N-1 row. Based on the above theory, in 2017, Shen et al. [26] experimentally demonstrated a cascaded mesh of 56 programmable MZIs with this triangular mesh, which improved the computational speed and power efficiency compared with that of traditional electronic processors.

## 3. Hardware Error

The N-dimensional unitary matrix implemented by MZI meshes has some deviations from the needed matrix in actual computation. On the one hand, the triangular mesh of the Reck design leads to an inconsistent optical loss of each output, and the larger mesh is, the greater the optical loss difference of each output, and the higher the total losses. On the other hand, more importantly, the beam splitter in MZI cannot implement the ideal 50:50 beam splitter due to the hardware error generated by the fabrication and the non-uniformity of the material, which leads to the deviation of the transmission matrix and affects the final computation.

The non-ideal 50:50 beam splitter transmission matrix can be expressed as follows:

(8) $$\left[\begin{array}{cc} cos\varphi & isin\varphi \\ isin\varphi & cos\varphi \end{array}\right]$$

where, when φ=π/4, it is the ideal 50:50 beam splitter; φ will generally deviate from π/4 for the non-ideal 50:50 beam splitter, which leads to the deviation of the MZI transmission matrix. For the transmission matrix of the beam splitter on the left side of the MZI and the beam splitter on the right side of the MZI, φ can be expressed as $\varphi_{1}$ and $\varphi_{2}$, respectively, and then MZI transmission matrix *S*′ can be expressed as

(9) $$S^{\prime }=\left[\begin{array}{cc} cos\varphi_{2} & isin\varphi_{2}\\ isin\varphi_{2} & cos\varphi_{2} \end{array}\right]\left[\begin{array}{cc} e^{i\theta } & 0\\ 0 & 1 \end{array}\right]\left[\begin{array}{cc} cos\varphi_{1} & isin\varphi_{1}\\ isin\varphi_{1} & cos\varphi_{1} \end{array}\right]\left[\begin{array}{cc} e^{i\varphi } & 0\\ 0 & 1 \end{array}\right]$$

(10) $$S^{\prime }=ie^{i\theta /2}\left[\begin{array}{cc} e^{i\varphi }\left(\begin{array}{l} cos\left(\varphi_{1}-\varphi_{2}\right)sin\left(\theta /2\right)-\\ icos\left(\varphi_{1}+\varphi_{2}\right)cos\left(\theta /2\right) \end{array}\right) & \left(\begin{array}{l} sin\left(\varphi_{1}+\varphi_{2}\right)cos\left(\theta /2\right)+\\ isin\left(\varphi_{1}-\varphi_{2}\right)sin\left(\theta /2\right) \end{array}\right)\\ e^{i\varphi }\left(\begin{array}{l} sin\left(\varphi_{1}+\varphi_{2}\right)cos\left(\theta /2\right)-\\ isin\left(\varphi_{1}-\varphi_{2}\right)sin\left(\theta /2\right) \end{array}\right) & -\left(\begin{array}{l} cos\left(\varphi_{1}-\varphi_{2}\right)sin\left(\theta /2\right)+\\ icos\left(\varphi_{1}+\varphi_{2}\right)cos\left(\theta /2\right) \end{array}\right) \end{array}\right]$$

Obviously, the MZI’s transmission matrix changes because of two non-ideal beam splitters, which is inconsistent with the transmission matrix in the ideal case, which leads to the deviation between the N-dimensional unitary matrix implemented by the MZI meshes and the theoretical one.

## 4. Error Correction

In terms of the hardware error in the N-dimensional unitary matrix transformation implemented by the MZI meshes introduced in Section 3, researchers have made a lot of effort. This section will introduce the main hardware error correction methods for (1) the entire MZI meshes and (2) a single MZI device.

### 4.1. The Entire MZI Meshes

#### 4.1.1. Rectangular Mesh

**Figure 3:**
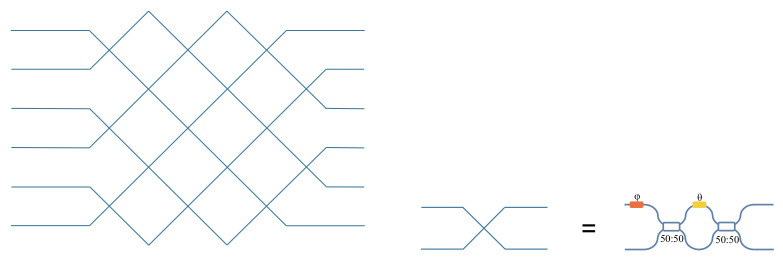
Rectangular mesh.

In 2016, Clements et al. [23] proposed a rectangular mesh, which was an improvement of the triangular mesh of Reck, as shown in Figure 3. Compared to the triangular mesh of Reck, the rectangular mesh has higher symmetry and a lower optical loss difference of each output. Additionally, the longest optical depth of the rectangular mesh is about half that of the triangular mesh, and the total optical loss is only half that of the triangular mesh.

**Figure 4:**
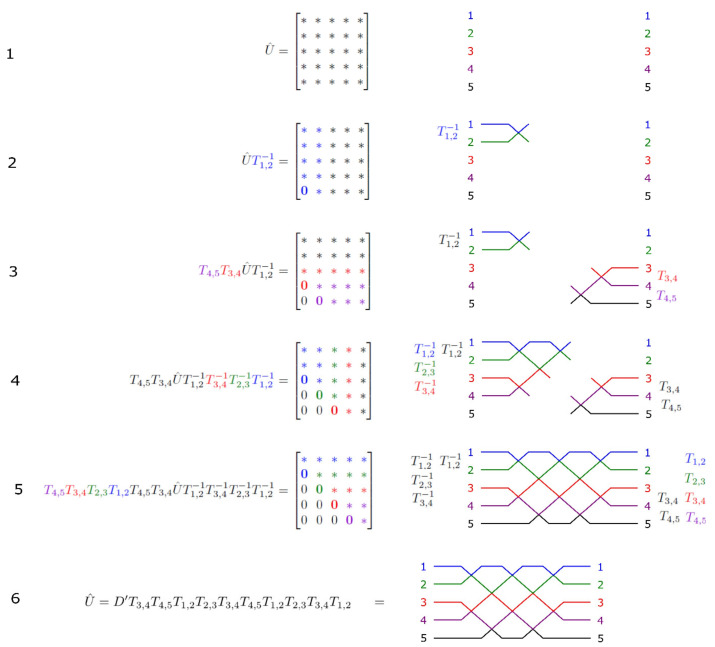
The decomposition method of the rectangular mesh. Reprinted under the terms of the CC-BY license [23]. Copyright 2016, Clements et al., published by Optica.

The improvement of the rectangular mesh is specifically manifested in the process of decomposition of the N-dimensional unitary matrix into a series of two-dimensional unitary matrices. The decomposition process does not reduce the dimensions by right multiplying *T*_n,m_(θ, φ), but by both right multiplying *T*_n,m_(θ, φ) and left multiplying *T*^−1^_n,m_(θ, φ), as shown in Figure 4 and seen in the following:

(11) $$T_{4,5}T_{3,4}T_{2,3}T_{1,2}T_{4,5}T_{3,4}UT_{1,2}^{-1}T_{3,4}^{-1}T_{2,3}^{-1}T_{1,2}^{-1}=D$$

that is,

(12) $$U=T_{3,4}^{-1}T_{4,5}^{-1}T_{1,2}^{-1}T_{2,3}^{-1}T_{3,4}^{-1}T_{4,5}^{-1}DT_{1,2}T_{2,3}T_{3,4}T_{1,2}$$

For a matrix *T*_n,m_(θ, φ) and a diagonal matrix *D*, there are matrix *T*^−1^_n,m_(θ, φ) and another diagonal matrix *D*′, with *T*^−1^_n,m_(θ, φ) *D*′ = *D T*_n,m_(θ, φ), and we obtain a right-multiplication dimensionality reduction operation similar to that in Equation (7).

(13) $$U=D^{\prime }T_{3,4}T_{4,5}T_{1,2}T_{2,3}T_{3,4}T_{4,5}T_{1,2}T_{2,3}T_{3,4}T_{1,2}$$

Shokraneh et al. [27] experimentally proved that due to the asymmetric distribution of MZIs in the triangular mesh, optical loss is greater on the triangular mesh than on the rectangular mesh in the computation process. They used a dataset that was perfectly classifiable to assess the classification performance of the two meshes in optical neural networks (ONN). Compared to the triangular mesh, the rectangular mesh is more phase-error-tolerant and loss-tolerant. Thus, the rectangular mesh is commonly adopted as the fundamental unit for constructing ONN in various research studies [28,29,30]. However, the beam splitter errors will still impact the computed result of the rectangular mesh.

#### 4.1.2. Fourier Structure

In order to solve hardware errors introduced by the non-ideal 50:50 beam splitter, Lopez-Pastor et al. [31] presented one kind of mesh composed of Fourier transforms and phase masks, which can implement the unitary matrix transformation of any finite dimension.

For an N-dimensional unitary matrix, the above Equation (13) can be written as

(14) $$U=D\prod_{i=1}^{N/2}\prod_{k=1}^{N/2-1}T_{2k}\left(\chi_{k}^{\left(i\right)},\eta_{k}^{\left(i\right)}\right)\prod_{j=1}^{N/2}T_{2j-1}\left(\theta_{j}^{\left(i\right)},\varphi_{j}^{\left(i\right)}\right),$$

$\prod_{k=1}^{N/2-1}T_{2k}\left(\chi_{k}^{\left(i\right)},\eta_{k}^{\left(i\right)}\right)$ and $\prod_{j=1}^{N/2}T_{2j-1}\left(\theta_{j}^{\left(i\right)},\varphi_{j}^{\left(i\right)}\right)$ can be decomposed by the diagonal matrix, permutation matrix and circulant matrix, and the decomposition of the unitary matrix can be obtained as follows:

(15) $$U=DG\left[\prod_{i=1}^{N/2}B^{\left(i\right)}A^{\left(i\right)}\right]G,$$

where *A*^(*i*)^ and *B*^(*i*)^ are

(16) $$A^{\left(i\right)}={\left\{E,G,H,p\left(\Gamma \left(\theta^{\left(i\right)}\right)\right),E,G\Gamma \left(\varphi^{\left(i\right)}\right)\right\}}_{F},$$

and,

(17) $$B^{\left(i\right)}={\left\{E,p\left(G\right),H,\Gamma \left(\chi^{\left(i\right)}\right),E,p\left(G\Gamma \left(\eta^{\left(i\right)}\right)\right)\right\}}_{F},$$

**Figure 5:**
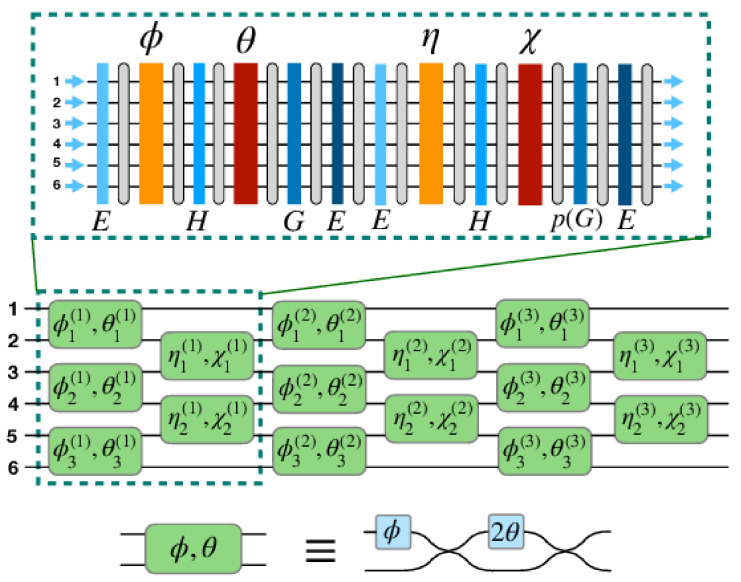
Mesh composed of Fourier transforms and phase masks. Reprinted under the terms of the OSA Open Access Publishing Agreement [31]. Copyright 2021, Lopez-Pastor et al., published by Opt. Express.

Finally, The N-dimensional unitary matrix, *U*(*N*), can be decomposed into a product of phase masks and Fourier transforms. As shown in Figure 5, the gray rounded rectangles represent the Fourier transforms, and the colored rectangles represent the phase-mask diagonal matrices. Only two diagonal matrices per layer (denoted by red and yellow rectangles) depend on the unitary matrix being implemented, while the rest (denoted by blue rectangles) are fixed. In this structure, the MZI of each layer in the mesh is decomposed into the form of Fourier transforms and phase masks, and the Fourier transforms can be implemented by MMI. This mesh avoids the use of a large number of beam splitters, reduces the source of error to a certain extent, and is more conducive to the realization of a large-scale mesh for optoelectronic computation. However, this decomposition method is not as simple and easy to implement as the triangular mesh and rectangular mesh methods are. Additionally, an even-dimension unitary matrix requires 6N DFTs (the discrete Fourier transform) and 6N + 1 controllable phase masks, and thus the size of the mesh will also significantly increase.

#### 4.1.3. Redundant Rectangular Mesh

**Figure 6:**
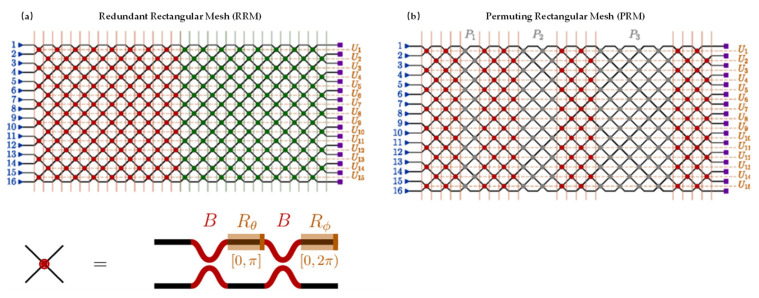
(**a**) Redundant rectangular mesh; (**b**) permuting rectangular mesh. Reprinted with permission from ref. [32]. Copyright 2019, APS.

Pai et al. [32] proposed two mesh improvements. The first is adding redundant tunable layers in the rectangular Mesh, called the redundant rectangular mesh (RRM), which can accelerate the optimization process of the mesh to implement the unitary matrix. The second is adding a redundant mesh composed of low-loss waveguide crossings or MZIs with fixed cross-state phase shifts, called the permuting rectangular mesh (PRM). As shown in Figure 6, Figure 6a is a schematic diagram of the RRM, where the green part represents the redundant tunable layers, and Figure 6b shows the PRM, where the gray part represents the additional low-loss waveguide crossings or MZIs with fixed cross-state phase shifts. This method of adding additional MZIs in the mesh increases the tunable degrees of freedom in the MZI’s mesh. For a given unitary matrix, there is a supersaturated implementation schemes. Some unitary matrices that cannot be realized due to MZI imperfections can be realized by new equivalent schemes brought by the extra degrees of freedom in the redundant mesh [33].

However, the redundant mesh increases the size of the MZI meshes, which inevitably leads to an increase in the optical loss of the entire mesh. In a small mesh, the increased optical loss is within an acceptable range, and the computational precision of the MZI meshes is better improved.

### 4.2. A Single MZI Device

In addition to the above improvements in the MZI’s mesh, more researchers focus on the improvement of a single MZI device. The improvement of a single MZI device is mainly carried out by adding redundant phase shifters and beam splitters to improve the tunable degrees of freedom of the MZI, so as to correct its hardware errors.

For a MZI with non-ideal beam splitters, where neither of the two beam splitters has an ideal 50:50 beam splitter ratio, the transmission matrix is given by Equation (10). When we input optical signal *E*_1_ from only one port of the MZI, the output optical powers *P_out_1_* and *P_out_2_* of the two output ports are

(18) $$P_{out\_1}={\left|E_{1}\right|}^{2}\left[cos^{2}\left(\varphi_{1}+\varphi_{2}\right)cos^{2}\left(\theta /2\right)+cos^{2}\left(\varphi_{1}-\varphi_{2}\right)sin^{2}\left(\theta /2\right)\right],$$

(19) $$P_{out\_2}={\left|E_{1}\right|}^{2}\left[sin^{2}\left(\varphi_{1}+\varphi_{2}\right)cos^{2}\left(\theta /2\right)+sin^{2}\left(\varphi_{1}-\varphi_{2}\right)sin^{2}\left(\theta /2\right)\right],$$

**Figure 7:**
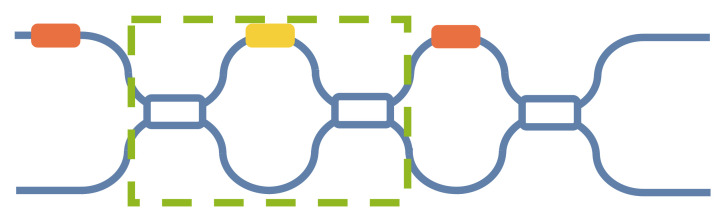
MZI architecture with a variable splitter as the front 3-dB splitter (MZI as a beam splitter in green frame).

When $\varphi_{1}+\varphi_{2}=\pi /2$, $P_{out\_1}={\left|E_{1}\right|}^{2}cos^{2}\left(\varphi_{1}+\varphi_{2}\right)cos^{2}\left(\theta /2\right)$, complete extinction can be achieved. Suzuki et al. [34] proposed a MZI architecture, replacing a beam splitter in the common MZI with a MZI, as shown in Figure 7. They adjusted this MZI as a 50:50 beam splitter so that its split ratio was complementary to that of another beam splitter to meet the above complete extinction conditions. Moreover, they demonstrated this 2 × 2 MZI with the highest extinction ratio (50.4 dB).

Although this architecture improves the extinction ratio of the MZI and corrects its transmission matrix to a certain extent, the correction of the transmission matrix is not complete. When complete extinction is achieved, MZI transmission matrix *S*″ can be expressed as

(20) $$S^{\prime \prime }=ie^{i\theta /2}\left[\begin{array}{cc} 2e^{i\varphi }\cos\varphi_{2}sin\varphi_{2}sin\left(\theta /2\right) & \left(cos\left(\theta /2\right)+i\cos\left(2\varphi_{2}\right)sin\left(\theta /2\right)\right)\\ e^{i\varphi }\left(cos\left(\theta /2\right)-icos\left(2\varphi_{2}\right)sin\left(\theta /2\right)\right) & -2\cos\varphi_{2}sin\varphi_{2}sin\left(\theta /2\right) \end{array}\right],$$

It is evident that the transmission matrix of MZI undergoes a slight correction, yet it still deviates from the ideal MZI transmission matrix’s *S* (Equation (3)). Consequently, errors may still arise during matrix computations, especially when dealing with large-scale MZI meshes.

**Figure 8:**
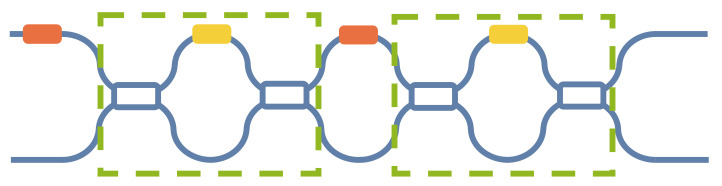
A double MZI architecture (MZI as a beam splitter in green frame).

Miller et al. [35] presented a double Mach–Zehnder interferometer (DMZI), as shown in Figure 8. Two MZIs were used as tunable beam splitters to replace the two splitters on the left and right of the common MZI, forming a new MZI architecture.

Now, we will illustrate the error correction process of DMZI. For instance, considering the MZI on the left, we should adjust its beam splitters to a 50:50 split. The transmission matrix of two beam splitters in this MZI can be expressed as *P_a_* and *P_b_*:

(21) $$P_{a}=\left[\begin{array}{cc} \sqrt{\frac{1}{2}-R_{a}} & i\sqrt{\frac{1}{2}+R_{a}}\\ i\sqrt{\frac{1}{2}+R_{a}} & \sqrt{\frac{1}{2}-R_{a}} \end{array}\right],$$

(22) $$P_{b}=\left[\begin{array}{cc} \sqrt{\frac{1}{2}-R_{b}} & i\sqrt{\frac{1}{2}+R_{b}}\\ i\sqrt{\frac{1}{2}+R_{b}} & \sqrt{\frac{1}{2}-R_{b}} \end{array}\right],$$

where, *R_a_* and *R_b_* represent errors of the left beam splitter and the right beam splitter, respectively. The transmission matrix, *P_L_*, of the MZI as a beam splitter can be expressed as

(23) $$P_{L}=\left[\begin{array}{cc} \sqrt{\frac{1}{2}-R_{b}} & i\sqrt{\frac{1}{2}+R_{b}}\\ i\sqrt{\frac{1}{2}+R_{b}} & \sqrt{\frac{1}{2}-R_{b}} \end{array}\right]\left[\begin{array}{cc} e^{i\theta } & 0\\ 0 & 1 \end{array}\right]\left[\begin{array}{cc} \sqrt{\frac{1}{2}-R_{a}} & i\sqrt{\frac{1}{2}+R_{a}}\\ i\sqrt{\frac{1}{2}+R_{a}} & \sqrt{\frac{1}{2}-R_{a}} \end{array}\right]\left[\begin{array}{cc} e^{i\varphi } & 0\\ 0 & 1 \end{array}\right],$$

This can be rewritten as

(24) $$P_{L}=\left[\begin{array}{cc} e^{i\theta }\sqrt{\alpha_{b}\alpha_{a}}-\sqrt{\beta_{b}\beta_{a}} & ie^{i\theta }\sqrt{\alpha_{b}\beta_{a}}+i\sqrt{\beta_{b}\alpha_{a}}\\ ie^{i\theta }\sqrt{\beta_{b}\alpha_{a}}+i\sqrt{\alpha_{b}\beta_{a}} & \sqrt{\alpha_{b}\alpha_{a}}-e^{i\theta }\sqrt{\beta_{b}\beta } \end{array}\right],$$

where, $\alpha_{b}=\frac{1}{2}-R_{b}$; $\beta_{b}=\frac{1}{2}+R_{b}$; $\alpha_{a}=\frac{1}{2}-R_{a}$; $\beta_{a}=\frac{1}{2}+R_{a}$. When we input optical signals, *E*_1_, from only one port of MZI, the output optical power, *P_out_*, of one of the two output ports is

(25) $$P_{out}={\left|E_{1}\right|}^{2}\left\{\frac{1}{2}+2\left[R_{a}R_{b}-\sqrt{\left(\frac{1}{4}-R_{a}^{2}\right)\left(\frac{1}{4}-R_{b}^{2}\right)}\times cos\theta \right]\right\},$$

Thus, if this MZI is used as a 50:50 beam splitter, $P_{3}=\frac{1}{2}{\left|E_{1}\right|}^{2}$; hence, from Equation (25)

(26) $$R_{a}R_{b}=\sqrt{\left(\frac{1}{4}-R_{a}^{2}\right)\left(\frac{1}{4}-R_{b}^{2}\right)}\times cos\theta ,$$

considering $cos^{2}\theta \leq 1$; hence, from Equation (26)

(27) $$R_{a}^{2}R_{b}^{2}\leq \left(\frac{1}{4}-R_{a}^{2}\right)\left(\frac{1}{4}-R_{b}^{2}\right),$$

which can be derived to give

(28) $$\left|R_{a}\right|\leq \sqrt{\frac{1}{8}}≃0.35\&\left|R_{b}\right|\leq \sqrt{\frac{1}{8}}≃0.35,$$

Therefore, the split ratios of the fabricated power from 85:15 to 15:85 can be compensated by adjusting the split ratio of the two tunable beam splitters back to 50:50. Two redundant beam splitters and two redundant phase shifters can completely correct the transmission matrix of MZI. Wilkes et al. [36] proposed a configuration algorithm for this DMZI, eventually achieving a 60 dB extinction ratio.

**Figure 9:**
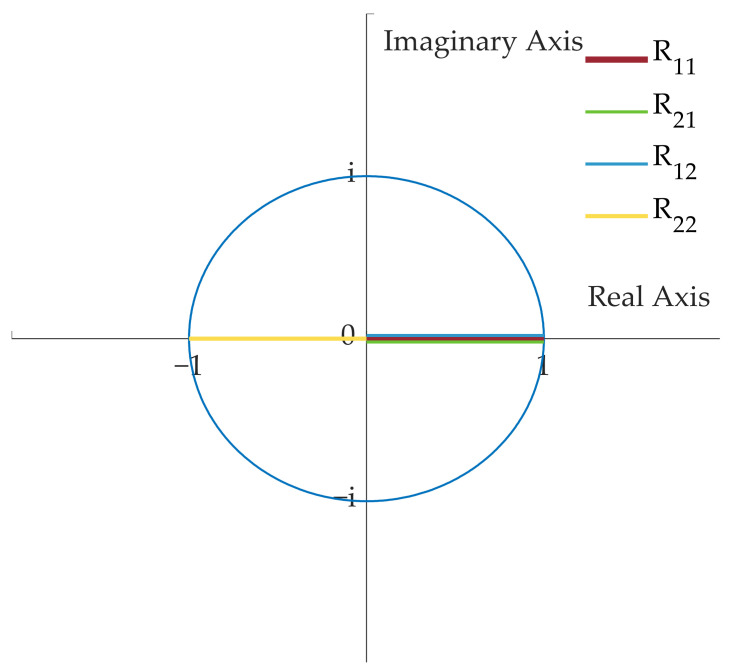
The corresponding output optical phase of the four elements in the matrix *R*.

However, the size of the MZI also increases significantly. The increase in optical loss brought about by redundant beam splitters and phase shifters is also a problem to be considered in large-scale MZI meshes. Moreover, the transmission matrix of a real MZI as a beam splitter is different from that of the ideal one; when the ideal MZI is used as a 50:50 beam splitter, its transmission matrix *R*, from Equation (3), is:

(29) $$R=\left[\begin{array}{cc} 1 & 1\\ 1 & -1 \end{array}\right],$$

where, $\;x=cos\left(\varphi_{1}-\varphi_{2}\right);y=cos\left(\varphi_{1}+\varphi_{2}\right);s=sin\left(\theta /2\right);c=cos\left(\theta /2\right)$. The corresponding output optical phase of the four elements in the matrix *R* can be represented on a complex plane, as shown in Figure 9.

**Figure 10:**
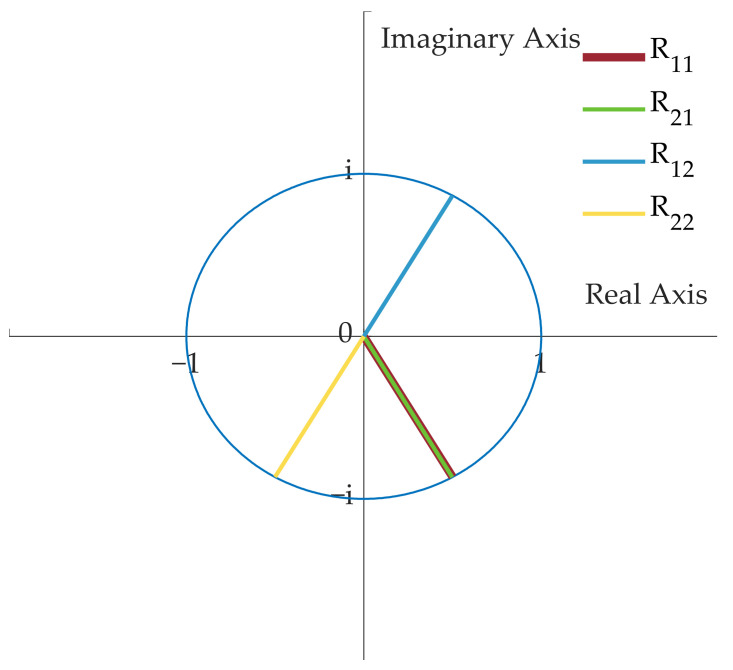
The corresponding output optical phase of the four elements in the matrix *R′*.

For a real MZI, when it is used as a 50:50 beam splitter, its transmission matrix *R*′, from Equation (10), is

(30) $$R^{\prime }=\left[\begin{array}{cc} sx-icy & c\sqrt{1-y^{2}}+is\sqrt{1-x^{2}}\\ c\sqrt{1-y^{2}}-is\sqrt{1-x^{2}} & -sx-icy \end{array}\right],$$

where, $\;x=cos\left(\varphi_{1}-\varphi_{2}\right);y=cos\left(\varphi_{1}+\varphi_{2}\right);s=sin{\left(\theta /2\right)}_{};c=cos\left(\theta /2\right)$. The corresponding output optical phase of the four elements in the matrix *R*′ can be represented on a complex plane, as shown in Figure 10.

**Figure 11:**
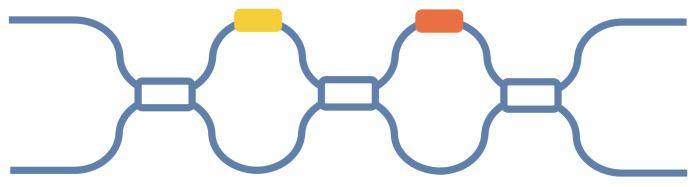
Three-splitter MZI.

**Figure 12:**
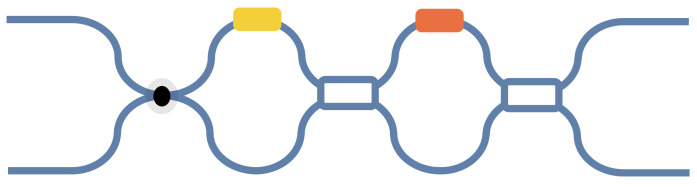
MZI + crossing.

By comparison with Figure 11 and Figure 12, it is evident that for a MZI with a beam splitter that is not an ideal 50:50 beam splitter, when it is used as a 50:50 beam splitter, the phase of the output optical signal will be altered, thus ultimately impacting the interference result in the subsequent optical path of the mesh and thus the final computed result.

Based on the above works, Hamerly et al. [20] proposed two MZI architectures (Figure 11 and Figure 12). One is of a three-splitter MZI, which can correct generic errors and achieve a full range of split ratios. To realize the full range of the split ratio, it changes the position of “forbidden regions” caused by the error of the beam splitter, which are some unitary matrices that cannot be implemented because of the beam splitter error. The “forbidden regions” are displaced away from the cross state, rather than being eliminated completely. However, similarly to the architecture proposed by Suzuki, it does not completely correct the transmission matrix of the MZI. Furthermore, this MZI architecture does not incorporate an external phase shifter. Therefore, when it is formed into a mesh, it cannot correct the phase error from the previous layeR′s MZIs.

The other one is MZI + crossing, which can only correct correlated device errors. Because the errors of the right and left beam splitters are consistent, the added cross waveguide rotates the “forbidden regions” by 180° to achieve complete extinction. Thus, this architecture has bandwidth tolerance. However, due to the errors of the right and left beam splitters being usually inconsistent, this architecture only exhibits good bandwidth tolerance but cannot correct the beam splitter error and eliminate limitations to matrix computation. Compared to the Suzuki design and Miller design, these two MZI architectures are smaller in size and do not add redundant phase shifters, but the hardware error correction is not good enough.

**Figure 13:**
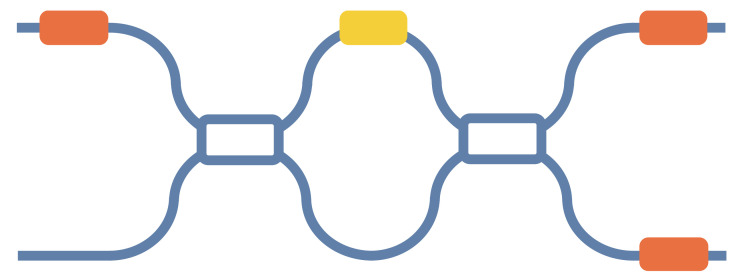
Bandyopadhyay et al.’s MZI architecture with a local error correction design.

Bandyopadhyay et al. [21] used redundant phase shifters to correct hardware errors and proposed a method of adding phase shifters to two ports of the output end of an MZI to locally correct the hardware error within an individual MZI. In this method, no additional beam splitters are added, and the increase in the size of a single device is small, as shown in Figure 13.

For the MZI with non-ideal 50:50 beam splitters, the transmission matrix *S′*, from Equation (10), can be rewritten as:

(31) $$S^{\prime }\left(\theta^{\prime },\varphi^{\prime }\right)=ie^{i\theta^{\prime }/2}\left[\begin{array}{cc} e^{i\varphi^{\prime }}\left(\begin{array}{l} cos\left(\varphi_{1}-\varphi_{2}\right)sin\left(\theta^{\prime }/2\right)-\\ icos\left(\varphi_{1}+\varphi_{2}\right)cos\left(\theta^{\prime }/2\right) \end{array}\right) & \left(\begin{array}{l} sin\left(\varphi_{1}+\varphi_{2}\right)cos\left(\theta^{\prime }/2\right)+\\ isin\left(\varphi_{1}-\varphi_{2}\right)sin\left(\theta^{\prime }/2\right) \end{array}\right)\\ e^{i\varphi^{\prime }}\left(\begin{array}{l} sin\left(\varphi_{1}+\varphi_{2}\right)cos\left(\theta^{\prime }/2\right)-\\ isin\left(\varphi_{1}-\varphi_{2}\right)sin\left(\theta^{\prime }/2\right) \end{array}\right) & -\left(\begin{array}{l} cos\left(\varphi_{1}-\varphi_{2}\right)sin\left(\theta^{\prime }/2\right)+\\ icos\left(\varphi_{1}+\varphi_{2}\right)cos\left(\theta^{\prime }/2\right) \end{array}\right) \end{array}\right],$$

To implement a desired unitary *S* (Equation (3)), we should find the $\theta^{\prime },\varphi^{\prime }$ of each $S^{\prime }\left(\theta^{\prime },\varphi^{\prime }\right)$ = *S*. The condition to each $S^{\prime }\left(\theta^{\prime },\varphi^{\prime }\right)$ = *S* produces the following expression for $\theta^{\prime }$:

(32) $$\theta^{\prime }=2\arcsin\sqrt{\frac{\sin^{2}\left(\theta /2\right)-\cos^{2}(\varphi_{1}+\varphi_{2})}{\cos^{2}(\varphi_{1}-\varphi_{2})-\cos^{2}(\varphi_{1}+\varphi_{2})}},$$

Therefore, the beam splitter errors restrict θ to the range

(33) $$2\left|\varphi_{1}+\varphi_{2}-\frac{\pi }{2}\right|<\theta <\pi -2\left|\varphi_{1}-\varphi_{2}\right|,$$

Assuming that θ is in this range, the transmission matrix’s $S^{\prime }\left(\theta^{\prime },\varphi^{\prime }\right)$, from Equation (31), can be rewritten as

(34) $$S^{\prime \prime }=ie^{i\theta^{\prime }/2}\left[\begin{array}{cc} e^{i\varphi^{\prime }}e^{i\delta_{a}}sin\left(\theta /2\right) & e^{i\delta_{b}}cos\left(\theta /2\right)\\ e^{i\varphi^{\prime }}e^{i\delta_{c}}cos\left(\theta /2\right) & -e^{i\delta_{d}}sin\left(\theta /2\right) \end{array}\right],$$

(35) $$=ie^{i\theta^{\prime }/2}\left[\begin{array}{cc} e^{i\delta_{b}} & 0\\ 0 & e^{i\delta_{d}} \end{array}\right]\left[\begin{array}{cc} e^{i\varphi^{\prime }}e^{i\left(\delta_{a}-\delta_{b}\right)}sin\left(\theta /2\right) & cos\left(\theta /2\right)\\ e^{i\varphi^{\prime }}e^{i\left(\delta_{c}-\delta_{d}\right)}cos\left(\theta /2\right) & -sin\left(\theta /2\right) \end{array}\right],$$

(36) $$=ie^{i\theta^{\prime }/2}\left[\begin{array}{cc} e^{i\delta_{b}} & 0\\ 0 & e^{i\delta_{d}} \end{array}\right]\left[\begin{array}{cc} e^{i\left(\varphi^{\prime }+\delta_{a}-\delta_{b}\right)}sin\left(\theta /2\right) & cos\left(\theta /2\right)\\ e^{i\left(\varphi^{\prime }+\delta_{a}-\delta_{b}\right)}cos\left(\theta /2\right) & -sin\left(\theta /2\right) \end{array}\right],$$

where $\delta_{a}$, $\delta_{b}$, $\delta_{c}$ and $\delta_{d}$ are phase errors the elements of $S^{\prime }\left(\theta^{\prime },\varphi^{\prime }\right)$, and for the unitary matrix requiring that $\delta_{a}+\delta_{d}=\delta_{b}+\delta_{c}$, we can correct those phase errors from Equation (36) to set $S^{\prime }\left(\theta^{\prime },\varphi^{\prime }\right)$ as equal to *S*. In accordance with Equation (36), the architecture shown in Figure 13 can be obtained. For different unitary matrices, corresponding error correction procedures must be implemented, and the correction method is complicated. It is important to note that this architecture can only correct the phase error. Although the transmission matrix is corrected, θ is restricted, meaning that some matrices cannot be implemented accurately. When θ is not in this range, there is a deviation between the desired matrix and the actual matrix.

## 5. Discussion

To overcome hardware errors in MZI meshes during the implementation of matrix computation, the primary strategies include increasing the tunable degrees of freedom of MZI meshes. This involves adding redundant beam splitters and phase shifters. However, incorporating a redundant mesh and improving a single MZI device may result in an increase in the size of the MZI mesh, as shown in Table 1. Therefore, it is crucial to develop a MZI mesh that can perform high-precision computation without increasing the device’s size to achieve large-scale matrix computation.

In 2001, a study report [37] was conducted on tunable multimode interference couplers (MMI). By changing the position of the four-fold images of MMI out of the phase shift of the four fields, the split ratio of the two-fold images was controlled, which was then realized in the InP material. In 2008, May-Arrioja et al. [38] used local electrical modulation to control the split ratio, and in addition, changed the phase of the two-fold images [39,40] to realize the arbitrary split ratio of the self-image. A thermally modulated MMI using polymer materials was also introduced [41]. In 2019, Perez et al. [42] reported a thermally modulated dual-drive directional coupler (DD-DC) and experimentally proved that it can realize an arbitrary split ratio. The size of the tunable beam splitters proposed in these studies is relatively large, and some beam splitters are not integrated on a silicon platform. However, these studies provide us with a novel perspective; by applying thermal or electrical modulation interference to the local optical field of the static beam splitter, we can also create a tunable beam splitter on a silicon platform without needing to increase its size.

**Figure 14:**
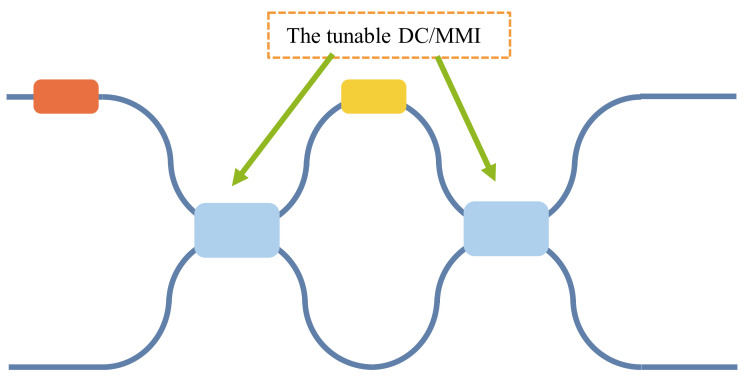
A new MZI architecture.

Here, we propose a new MZI architecture, where we replace the 50:50 beam splitter in the common MZI with a tunable DC/MMI that takes into account hardware error correction and mesh size, as shown in Figure 14. This improves the computational precision of the MZI’s mesh without increasing its size. By adjusting the split ratios of the tunable DC/MMI, we can eliminate its split ratio deviations from 50:50. For instance, for a tunable MMI, when the phase of optical fields at the position of the four-fold images in the multimode interferometer is altered, it affects the interference results of the optical fields, culminating in changes in the intensity of the two images at the two-fold image position. This enables the adjustment of the beam split ratios. The tunable MMI has the theoretical capability to adjust split ratios from 100:0 to 0:100, enabling the adjustment of any fabricated power split ratios in the physical 2 × 2 MMI to the ideal 50:50 split. This correction completely eliminates beam splitter errors and enables high-precision MZI-based matrix computation.

## 6. Conclusions

To summarize, this paper introduces the method of using a MZI’s mesh to implement any finite dimensional unitary matrix transformation, along with an analysis of the effects of hardware errors during matrix computation based on the MZI. Addressing MZIs’ hardware errors is crucial to achieving large-scale matrix computation. To eliminate the hardware error of an MZI, various improvement works have been carried out on the entire MZI mesh and a single MZI device. It is important to note that correcting these errors can lead to an increase in an MZI’s mesh size, which is also a significant concern. The trade-off between hardware error correction and mesh size requires more innovative works in artificial intelligence, materials, optics, device manufacturing and other related fields.

In this paper, a new MZI architecture is proposed which replaces the 50:50 beam splitter in the common MZI with the tunable DC/MMI. This MZI is designed to take into account both hardware error correction and mesh size concerns. The tunable beam splitter-based MZI provides a new approach for more accurate large-scale matrix computation based on the MZI’s mesh.

## Figures and Tables

**Table 1:** Characteristics of major MZI schemes.

Architecture	Number of Beam Splitters	Number of Phase Shifts	Size	Hardware Error Correction
The common MZI	2	2	1	none
Suzuki	3	3	1.5	+++
Miller	4	4	2	++++
3-splitter MZI	3	2	1.2	++
MZI + crossing	2	2 + 1 crossing	1.2	+
Bandyopadhyay	2	4	1.2	++

## Data Availability

No new data were created or analyzed in this study. Data sharing is not applicable to this article.
